# Readability of Information Generated by ChatGPT for Hidradenitis Suppurativa

**DOI:** 10.2196/55204

**Published:** 2024-08-14

**Authors:** Lauren Gawey, Caitlyn B Dagenet, Khiem A Tran, Sarah Park, Jennifer L Hsiao, Vivian Shi

**Affiliations:** 1 University of Arkansas for Medical Sciences Little Rock, AR United States; 2 College of Medicine The University of Arizona Tucson, AZ United States; 3 Department of Dermatology University of Arkansas for Medical Sciences Little Rock, AR United States; 4 David Geffen School of Medicine University of California Los Angeles Los Angeles, CA United States; 5 Department of Dermatology University of Southern California Los Angeles, CA United States

**Keywords:** hidradenitis suppurativa, ChatGPT, Chat-GPT, chatbot, chatbots, chat-bot, chat-bots, machine learning, ML, artificial intelligence, AI, algorithm, algorithms, predictive model, predictive models, predictive analytics, predictive system, practical model, practical models, deep learning, patient resources, readability

## Introduction

ChatGPT is an artificial intelligence (AI) language model that has emerged as a resource for patient education, with over 100 million general users worldwide [1]. Despite its popularity, the readability of information provided by ChatGPT on dermatological conditions, such as hidradenitis suppurativa (HS), has yet to be explored. Patients with HS wait an average of 7 years after their initial symptoms appear to seek medical attention, which is largely attributed to insufficient awareness of the condition [2]. Effective patient education is vital for informed decision-making and self-management of medical conditions. The American Medical Association and the National Institutes of Health recommend that patient educational materials should be written at a sixth- and eighth-grade reading level, respectively [3]. This study aimed to assess the readability of ChatGPT-generated responses in comparison to established HS educational materials and web-based resources.

## Methods

We compared the readability of responses to frequently asked questions from the HS Foundation (HSF), HS Patient Guide (HSPG) [4], and ChatGPT-3.5, along with HS-related websites (Google, Yahoo, and Bing were searched using the term “hidradenitis suppurativa”). The top 50 web pages from each search engine were reviewed, of which, 55 met inclusion criteria for further analysis. Readability was determined by average readability grade level and Flesch Reading Ease, which is scored from 0 to 100, with a higher score indicating that the material is easier to read. These readability formulas take into account the number of characters, syllables, words, and sentences to determine their score. Lexical density—a measure of linguistic complexity—and other text readability metrics were also recorded. While reviewers did not directly participate in the scoring process, the use of standardized software from online-utility.org facilitated objective evaluations aligned with established criteria for readability assessment. The 2-tailed Student *t* test was used for bivariate analysis, with significance set at *P*<.05.

## Results

**Figure 1:**
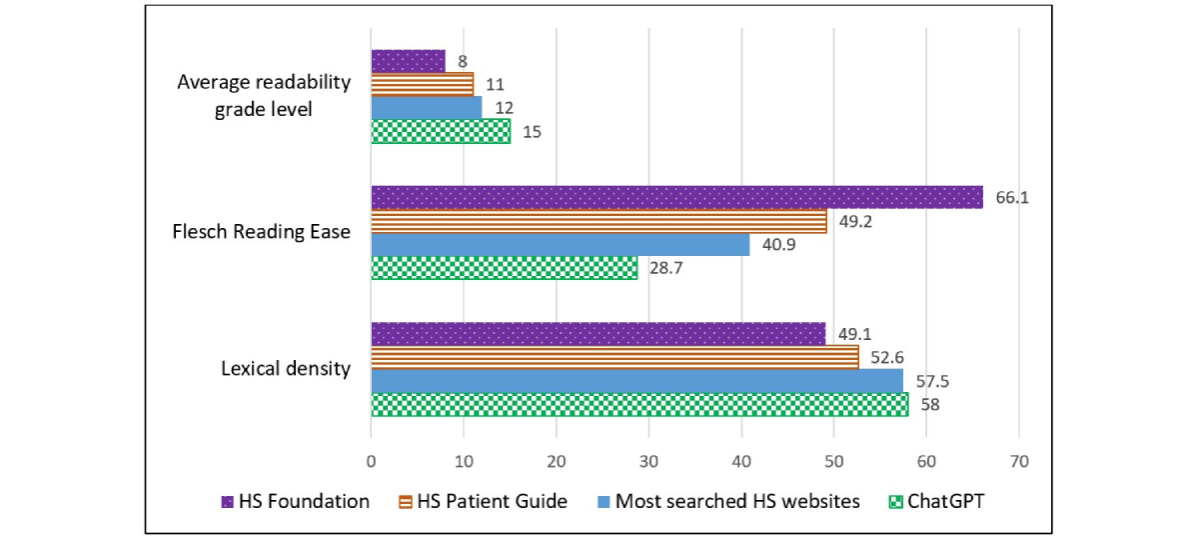
Readability of information for patients with hidradenitis suppurativa (HS) based on the average readability grade level, Flesch Reading Ease, and lexical density. The average readability grade level is calculated by averaging the Flesch Kincaid Grade Level, Gunning Fog Index, Simple Measure of Gobbledygook index, Coleman–Liau index, and automated readability index scores. Flesch Reading Ease is scored between 0 and 100, with a higher score indicating that the article is easier to read. Lexical density estimates linguistic complexity in a composition from the functional words (grammatical units) and content words (lexical units), calculated by comparing the ratio of lexical items to the total number of words.

**Figure 2:**
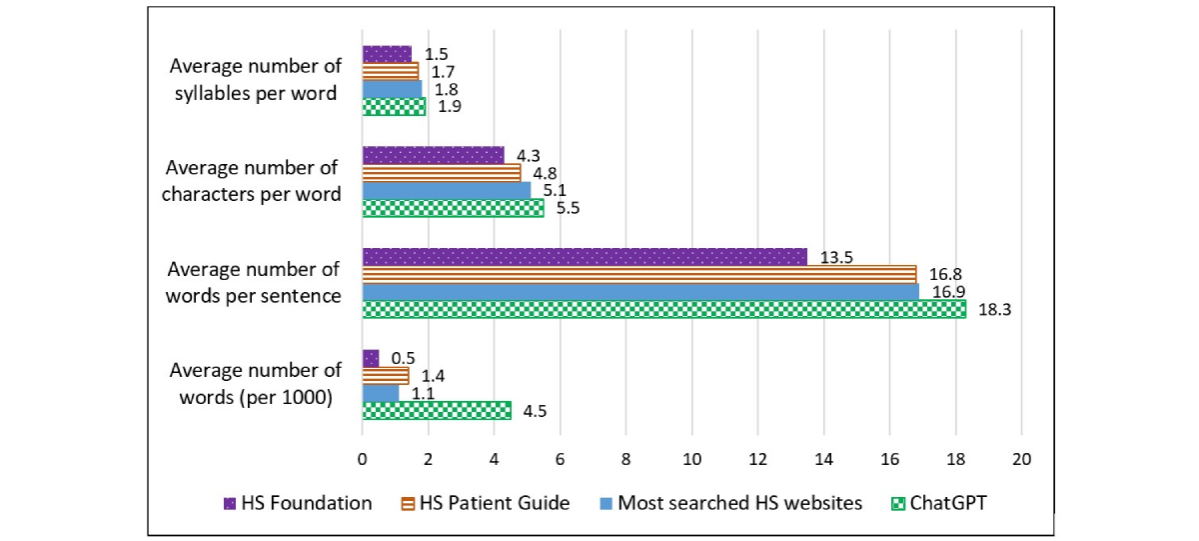
Text readability metrics of information for patients with hidradenitis suppurativa (HS). These values represent an average of text readability metrics for each specified source.

ChatGPT-generated responses had an average readability grade level of 15.0, which was significantly higher than that of the HSF (8.0), the HSPG (11.0), and HS-related websites (12.0; *P*<.001). Flesch Reading Ease was significantly lower for ChatGPT-generated responses (28.7) than for the HSF (66.1), the HSPG (49.2), and HS-related websites (40.9; *P*<.001; Figure 1). Both ChatGPT and HS-related websites had a higher lexical density of 58.0 and 57.47 respectively, indicating higher linguistic complexity than that for the HSF (49.1) and the HSPG (52.6; Figure 2).

## Discussion

Our results show that ChatGPT-generated responses were 7-9 grade levels above the recommended reading level and had a higher linguistic complexity than other HS-related web-based resources. These findings underscore the limitations of ChatGPT as a patient resource for HS, as the higher reading level and linguistic complexity of ChatGPT could hinder patient comprehension. The potential of AI-driven resources, such as ChatGPT, to transform health care communication hinges on their ability to align with recommended readability standards. One study showed that when prompting AI to convert patient educational material to an easier grade level, AI could improve the readability of input material [5]. However, without prompting, the baseline reading level of ChatGPT-generated information is much higher than is recommended for patient educational materials. It is important to note that the practice of prompting AI systems for readability adjustments is currently not commonplace among the general public user base. As AI integration becomes more commonplace, future studies can explore and compare the effectiveness of prompting strategies to make consistent adjustments in readability. Educating health care providers about the availability of options to prompt ChatGPT responses for enhanced readability can allow them to counsel their patients on adjusting readability levels that are most suitable for their preferences.

While the readability formulas used in this study offer a useful quantitative measure of text complexity, they focus primarily on surface-level features such as sentence length and syllable count, neglecting the structural complexity of texts, such as coherence, organization, and language context, which also influence readability. Additionally, AI-generated texts may exhibit variations in tone, style, and content that traditional readability formulas may struggle to evaluate accurately.

Future directions should work toward improving not only the readability of AI, but also the quality and accuracy of generated information. The findings of this study serve as a foundational reference for future AI resource development in dermatology.

## Abbreviations

AI: artificial intelligence
HS: hidradenitis suppurativa
HSF: Hidradenitis Suppurativa Foundation
HSPG: Hidradenitis Suppurativa Patient Guide
